# Kv1.3 Channel as a Key Therapeutic Target for Neuroinflammatory Diseases: State of the Art and Beyond

**DOI:** 10.3389/fnins.2019.01393

**Published:** 2020-01-14

**Authors:** Xiaoli Wang, Guoyi Li, Jingkang Guo, Zhiping Zhang, Shuzhang Zhang, Yudan Zhu, Jiwei Cheng, Lu Yu, Yonghua Ji, Jie Tao

**Affiliations:** ^1^Department of Neurology and Central Laboratory, Putuo Hospital, Shanghai University of Traditional Chinese Medicine, Shanghai, China; ^2^Institute of Biomembrane and Biopharmaceutics, Shanghai University, Shanghai, China; ^3^Xinhua Translational Institute for Cancer Pain, Shanghai, China; ^4^Putuo Clinical Medical School, Anhui Medical University, Shanghai, China

**Keywords:** Kv1.3, ShK, neuroinflammatory disease, multiple sclerosis, stroke, epilepsy, Alzheimer’s disease, Parkinson’s disease

## Abstract

It remains a challenge for the effective treatment of neuroinflammatory disease, including multiple sclerosis (MS), stroke, epilepsy, and Alzheimer’s and Parkinson’s disease. The voltage-gated potassium Kv1.3 channel is of interest, which is considered as a novel therapeutic target for treating neuroinflammatory disorders due to its crucial role in subsets of T lymphocytes as well as microglial cells. Toxic animals, such as sea anemones, scorpions, spiders, snakes, and cone snails, can produce a variety of toxins that act on the Kv1.3 channel. The *Stichodactyla helianthus* K^+^ channel blocking toxin (ShK) from the sea anemone *S. helianthus* is proved as a classical blocker of Kv1.3. One of the synthetic analogs ShK-186, being developed as a therapeutic for autoimmune diseases, has successfully completed first-in-man Phase 1 trials. In addition to addressing the recent progress on the studies underlying the pharmacological characterizations of ShK on MS, the review will also explore the possibility for clinical treatment of ShK-like Kv1.3 blocking polypeptides on other neuroinflammatory diseases.

## Introduction

Kv1.3 is a classical Shaker-type potassium channel with six transmembrane segments (Wulff and Zhorov, 2008), discovered in one of the non-excitable cells, T lymphocytes (Chiang et al., 2017), in the early 1980s. It is worth mentioning that Kv1.3 is the first K^+^ channel to be identified outside electrically excitable tissues. Kv1.3 in T lymphocytes is responsible for controlling the membrane potential which is critical for the activation of these immune cells (Veytia-Bucheli et al., 2018). Several studies have confirmed that Kv1.3 is highly expressed in macrophages, microglia, and TEM cells, suggesting that Kv1.3 plays a crucial role in immune and inflammatory responses to human diseases such as multiple sclerosis (MS), rheumatoid arthritis, Type 1 diabetes, and asthma (Toldi et al., 2010; Huang et al., 2017; Tanner et al., 2017; Zhou et al., 2018). In these conditions, the expression of Kv1.3 channels is significantly elevated (Rangaraju et al., 2009), which is beneficial to define the role of Kv1.3 in autoimmune diseases as well as to clarify the significance of developing Kv1.3 blocker drugs.

**FIGURE 1:**
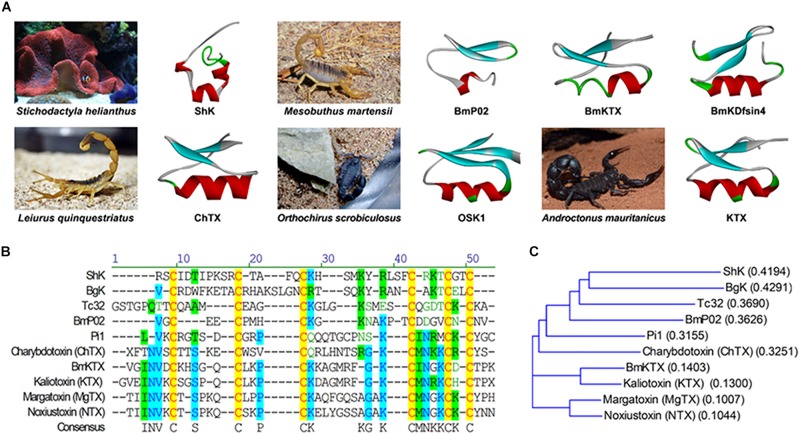
The structures of Kv1.3 blocking toxin peptides. **(A)** The sea anemone toxin ShK (PDB: 1ROO) is isolated and purified from *Sidirodromi hellinikou*. The scorpion toxins BmP02 (PDB: 1DU9), BmKTX (PDB: 1BKT), and scorpion defensin BmKDfsin4 (using micasin as a template, PDB: 2LR5) are isolated and purified from *Mesobuthus martensii*. The scorpion toxin ChTX (PDB: 2CRD) is isolated and purified from *Leiurus quinquestriatus*. The scorpion toxin OSK1 (PDB: 1SCO) is isolated and purified from *Orthochirus scrobiculosus*. The scorpion toxin KTX (PDB: 2KTX) is isolated and purified from *Androctonus mauritanicus*. The three-dimensional structure data of the toxin polypeptide in the figure refers to the PDB. **(B)** Multiple sequence alignment of ShK and ShK-like Kv1.3 blockers from sea anemone or scorpion venom. Conserved cysteines formatting intrachain disulfide bonds are in red and shadowed in yellow; residues conserved in most of the peptides are shadowed in blue; residues with same charge in most of the peptides are shadowed in green. The species of toxins acting on Kv1.3 are mentioned above, except for Tc32 isolated from *Tityus cambridgei*; Pi1 isolated from *Pandinus imperator*; Kaliotoxin isolated from *Androctonus mauritanicus*; margatoxin isolated from *Centruroides margaritatus*; noxiustoxin isolated from *Centruroides noxius*. **(C)** A guide tree is constructed by ALIGNX, a component of the VECTOR NTI 11.0 software suite. Scores in the brackets are based on the identity of the amino acids chemical properties.

Toxin peptides from natural toxic animals are the largest family of ion channel blockers. They are becoming the medicinal arsenal for the treatment of various diseases including neuroinflammatory disorders. Toxin peptides targeting Kv channels have been isolated and identified so far (Zhao et al., 2015). Toxins derived from scorpion, sea anemone, snakes, and other animals are found to block Kv1.3 with different affinity and selectivity (Table 1 and Figure 1). The reference data for the described toxin polypeptides are from the Protein Data Bank archive^1^ (PDB). One of the most representative Kv1.3 blockers is sea anemone toxin peptides. Sea anemones have a rich source of peptide toxins acting on ion channels, which are presumably present in special spiny organelles (nematodes) (Madio et al., 2019). These toxins can be used to catch prey as well as defend against predators (Prentis et al., 2018). Up to now, a number of toxins have been isolated and purified from the venom of various species of sea anemones. Most of sea anemone toxins can be divided into three types: porous soluble cytins which can be inhibited by sphingolipin (Anderluh and Macek, 2002); neurotoxins acting on voltage-gated Na^+^ channels (Norton, 1991); and the toxins acting on the voltage-gated Kv1 channels (Norton, 1991; Castaneda et al., 1995), which have different molecular weights. Among these toxins, both Na^+^ and K^+^ channel peptide toxins are considered as useful tools for investigating both the structure and function of channels, even developing potential drugs for the treatment of ion channelopathies because of the high specific and affinity to target channels.

**TABLE 1:** Examples of ShK-related natural venom peptides acting on Kv1.3.

**Peptide**	**Species**	**Number of residues**	**Disulfide pattern**	**IC_50_ or *K*_d_**	**Related diseases**
Sea anemone toxin ShK	*Stichodactyla helianthus*	35	C1–C6, C2–C4, C3–C5	133 pM (Castaneda et al., 1995)	Psoriasis (Tarcha et al., 2017); multiple sclerosis (Beeton et al., 2005); ischemic stroke (Peng et al., 2014); Alzheimer’s disease (Peng et al., 2014)
Sea anemone toxin BgK	*Bunodosoma granuliferum*	37	C1–C6, C2–C4, C3–C5	3.6 ± 0.6 nM (Aneiros et al., 1993)	Perhaps multiple sclerosis (Beraud et al., 2006)
Scorpion toxin BmP02	*Mesobuthus martensii*	28	C1–C4, C2–C5, C3–C6	7.0 ± 0.6 nM (Zhu et al., 2012)	Perhaps multiple sclerosis (Wu et al., 2016)
Scorpion toxin BmKTX	*Mesobuthus martensii*	37	C1–C4, C2–C5, C3–C6	200 pM (Renisio et al., 2000)	Multiple sclerosis (Devaux et al., 2004); Alzheimer’s disease (Norton and Chandy, 2017); Parkinson’s disease (Tubert et al., 2016)
Scorpion defensin BmKDfsin4	*Mesobuthus martensii*	37	NR	510.2 nM (Meng et al., 2016)	NR
Scorpion toxin OSK1	*Orthochirus scrobiculosus*	38	C1–C4, C2–C5, C3–C6	14.0 ± 1.0 pM (Mouhat et al., 2005)	Multiple sclerosis (Tegla et al., 2011); Alzheimer’s disease (Tarcha et al., 2017)
Scorpion toxin Kaliotoxin (KTX)	*Androctonus mauritanicus*	38	C1–C4, C2–C5, C3–C6	0.41 ± 0.23 nM (Gairi et al., 1997)	NR
Scorpion toxin Charybdotoxin (ChTX)	*Leiurus quinquestriatus*	37	C1–C4, C2–C5, C3–C6	0.71 ± 0.19 nM (Gimenez-Gallego et al., 1988)	Multiple sclerosis (Hu et al., 2007)
Scorpion toxin Maurotoxin (MTX)	*Scorpio maurus*	34	C1–C5, C2–C6, C3–C4, C7–C8	180 nM (Kharrat et al., 1997)	Perhaps multiple sclerosis (Jensen et al., 2002)
Scorpion toxin Noxiustoxin (NTX)	*Centruroides noxius*	39	C1–C4, C2–C5, C3–C6	0.31 ± 0.12 nM (Sitges et al., 1986)	NR
Scorpion toxin Pi1	*Pandinus imperator*	35	C1–C5, C2–C6, C3–C7, C4–C8	11.4 nM (Péter et al., 2000)	NR
Scorpion toxin Vm24	*Vaejovis smithi*	36	C1–C5, C2–C6, C3–C7, C4–C8	2.9 pM (Gurrola et al., 2012)	NR
Worm peptide AcK1	*Ancylostoma caninum*	51	C1–C6, C2–C4, C3–C5	266 nM (Chhabra et al., 2014)	NR
Snake toxin BF9	*Bungarus fasciatus*	65	C1–C6, C2–C4, C3–C5	120 nM (Yang et al., 2014)	Perhaps ischemic stroke (Ding et al., 2018)

*IC_50_, half maximal inhibitory concentration; *K*_*d*_, dissociation constant; NR, not reported.*

K^+^ channel peptide toxins could be classified into three types based on the structural and functional differences. Type 1 potassium channel toxins include five members, ShK from *Stichodactyla helianthus* (Castaneda et al., 1995), BgK from *Bunodosoma granulifera* (Cotton et al., 1997), AeK from *Actinia equina* (Minagawa et al., 1998), and HmK from *Heteractis magnifica* (Gendeh et al., 1997), which can block Kv1 (Shaker) potassium channels. Type 2 potassium channel toxins include AsKC 1–3 (kalicludines 1–3) (Schweitz et al., 1995), which block Kv1 channels much less effectively than Type 1 toxins. Furthermore, Type 3 potassium channel toxins include BDS-I and II from *Anemonia sulcata* which can specific block Kv3.4 channels and APETx1 from *Anthopleura elegantissima* (Diochot et al., 1998, 2003). The alignment of homologous sequence reveals that ShK has low homology with other K^+^ channel blocking peptides, except for BgK from the sea anemone *B. granulifera* (Castaneda et al., 1995). The alanine-scanning experiment identifies that three residues, Ser-20, Lys-22, and Tyr-23, are essential for ShK (Pennington et al., 1996) to bind K^+^ channels from rodent brain. Interestingly, these residues are also conserved in other Type 1 toxins. Especially, the dyad (Lys–Tyr) of the three residues is recently considered as the key player for binding potassium channels (Honma and Shiomi, 2006). In order to design the potential drugs targeting Kv1.3-related immune diseases with higher selectivity, the original toxin was engineered with chemical modification or site mutant genesis techniques. As a representative K^+^ blocker, ShK has been receiving great attentions because of its higher affinity on Kv1.3 than other toxins previously described. At the same time, it exhibits effective blocking of other Kv channel isoforms in various important tissues with the affinity of pM concentration, such as Kv1.1 (cardiac), Kv1.4 (brain), and Kv1.6 (brain) (Beeton et al., 2011). Therefore, it is of importance to develop more selective analogs for Kv1.3 (Chi et al., 2012).

Due to the affinity of ShK for other Kv channel subtypes, the development of ShK analogs with higher selectivity for Kv1.3 has been promoted. The mimetic ShK-Dap22, in which Lys22 was replaced by a shorter, positively charged, non-natural amino acid diaminopropionic acid (Dap) (Middleton et al., 2003). Compared with ShK, it can inhibit Kv1.3 in sub-nanomolar concentration *in vitro* and has lower toxicity. ShK-170, it contains an L-phosphotyrosine attached via an aminoethyloxyethyloxy-acetyl (Aeea) linker to the α-amino group of Arg. To stabilize the C-terminus of ShK-170 replaced the C-terminal carboxyl with an amide to minimize digestion by carboxypeptidases. The novel analog ShK-186 retains the selectivity and potency profile of ShK-170 (Chi et al., 2012). ShK-186 which had a 100-fold improvement of selectivity for Kv1.3 over Kv1.1, and 1000-fold over Kv1.4 as well as Kv1.6 (Pennington et al., 2009). ShK-186 and its analogs had good therapeutic effects on animal models of human autoimmune diseases such as MS and rheumatoid arthritis (Beeton et al., 2001). Preclinical testing of ShK-186 show favorable results both in rats and monkeys (Tarcha et al., 2012). Unexpectedly, ShK-186 was found to have a long half-life through the sub-cutaneous injection, which revealed the sustained concentration at pM levels in plasma, resulting in a prolonged therapeutic efficacy (Tarcha et al., 2012). ShK-186 as a preclinical drug, which is also known as dalazatide, completed Phase 1a and 1b trials in 2016. The Phase 1b trial in mild-to-moderate plaque psoriasis patients showed that dalazatide was well tolerated and reduced psoriatic skin lesions (Tarcha et al., 2017). Up to now, dalazatide is being advanced as a treatment for various autoimmune diseases, including inclusion body myositis, lupus, ANCA vasculitis, MS, psoriasis, psoriatic arthritis, rheumatoid arthritis, Type 1 diabetes, and inflammatory bowel diseases (Chandy and Norton, 2017; Liao et al., 2019).

In addition, Kv1.3 could even be inhibited by scorpion toxins ranging from nanomolar to picomolar, including noxiustoxin (NTX) (Drakopoulou et al., 1995), charybdotoxin (ChTX) (Drakopoulou et al., 1995), margatoxin (MgTX), *Orthochirus scrobiculosus* toxin 1 (OSK1), kaliotoxin, agitoxin-2, hongotoxin, and anuroctoxin (Bhuyan and Seal, 2015; Schwartz et al., 2017). ChTX, a 37-residue polypeptide present in the venoms of the scorpion *Leiurus quinquestriatus* var. MgTx is a 39 amino acid peptide derived from *Centruroides margaritatus* with an IC_50_ of 11.7 pM against Kv1.3. HsTX1, from *Heterometrus spinifer*, is a 34-residue toxin peptide, C-terminally amidated peptide cross-linked by four disulfide bridges (Rashid et al., 2014). BmKTX, from *Buthus martensii*, was modified at three residues to create ADWX-1, which blocked Kv1.3 with 2 pM affinity (Han et al., 2008). OSK1 is a 38-residue toxin cross-linked by three disulfide bonds isolated from the venom of the Asian scorpion *O. scrobiculosus*. Bs6 toxin is a short-chain neurotoxin of 38 amino acid residues isolated from *Buthus sindicus* (Kohl et al., 2015). These toxins are typical Kv1.3 inhibitors and are also considered to have potential for the treatment of neuroinflammatory diseases (Table 1 and Figure 1).

We note that Kv1.3 plays a key role in physiological processes of T lymphocytes and microglial cells and is considered as a drug-target for neuroinflammatory diseases. Therefore, in this review, we also discuss the potential utility of ShK and other Kv1.3 blocking peptides in neuroinflammatory disorders therapy.

## S*h*K and Its Analogs as a Potential Therapeutic Agent for Neuroinflammatory Diseases

### Multiple Sclerosis

Multiple sclerosis, a demyelination disease of the central nervous system (CNS) which is caused by a variety of factors including autoimmunity, genetics, environmental factors, and individual susceptibility factors (Kaminska et al., 2017). The occurrence of MS is often accompanied by the destruction of the blood–brain barrier (BBB) and the infiltration of the CNS by reactive T-cell (Huang et al., 2017). Some studies have shown that the permeability of BBB is increased at the early stage of MS (Kirk et al., 2003; Ortiz et al., 2014). In animal model of experimental autoimmune encephalomyelitis (EAE), the results show that BBB tight junctions *in vivo* and *in vitro* were destroyed. It might be induced by the increase of inflammatory interleukin-17 (IL-17), which is produced from activated Th17 cells (Kebir et al., 2007; Peelen et al., 2011). In addition, astrocytes also play an important role in MS (Nair et al., 2008; Brosnan and Raine, 2013). Activated astrocytes increase BBB permeability and promote T cell entry into the CNS by withdrawing the foot around their blood vessels (Brosnan and Raine, 2013).

**FIGURE 2:**
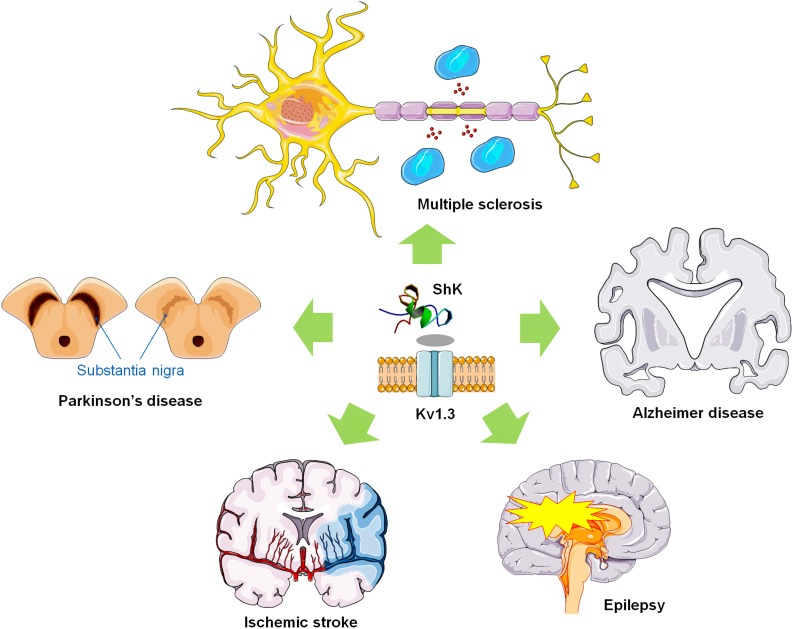
ShK and Kv1.3 blocking polypeptides as potential therapeutic agents for neuroinflammatory diseases. ShK and Kv1.3 toxins might be used to treat multiple sclerosis, and other neuroinflammatory disorders via inhibiting Kv1.3 expressed in T lymphocytes as well as microglial cells.

Based on the expression of the chemokine receptor CCR7 and phosphatase CD45RA, memory T lymphocytes can be divided into two subsets of central memory T cells (TCM) and effector memory T cells (TEM) (Pucca et al., 2016). TEM cells (CCR7-CD45RA-) rapidly enter the inflamed tissue, producing a large number of pro-inflammatory cytokines such as interferon-γ (IFN-γ) and IL-4, and exhibit immediate effector function. The Kv1.3 was first discovered in human T cells in 1984 (DeCoursey et al., 1984). Accumulated data for Kv1.3 showed higher expression levels in myelin-reactive T cells from the peripheral blood (PB) of MS patients compared to healthy controls (Wulff et al., 2003). Also in animal model of EAE, it has been confirmed that expression of Kv1.3 is significantly elevated (Rus et al., 2005) (Figure 2). These studies provide further rationale for the use of specific Kv1.3 antagonists in MS therapy.

Kv1.3 blocks membrane depolarization and maintains the driving force for Ca^2+^ entry by effluxing K^+^, which in turn participates in T cell activation, and Ca^2+^ activation signaling cascade, leading to T cell proliferation and cytokine production (Wulff et al., 2009; Bozic et al., 2018). These findings suggest Kv1.3 to be a valuable therapeutic target for immunosuppression in MS and EAE (Beeton et al., 2006; Wulff and Zhorov, 2008).

Kv1.3 channel blockers have been found to alleviate disease symptoms in animal autoimmune diseases, chronic inflammatory diseases, and metabolic disease models without obvious side effects (Perez-Verdaguer et al., 2016). More importantly, positive results have been shown in preclinical trials (Prentis et al., 2018), for example, improving the visual field and motor skills of most MS patients (Beeton and Chandy, 2005; Perez-Verdaguer et al., 2016). Therefore, Kv1.3 channel blockers have the potential to be developed as effective drugs for the treatment of MS and EAE (Rangaraju et al., 2009).

Blockers of Kv1.3 and other potassium channels have been found in the venom of numerous animals, including the venom of anemone (Wulff et al., 2019). In 1995, an effective K^+^ channel blocker was extracted from the sea anemone (*S. helianthus*) by Castaneda et al. (1995) and then named it ShK (Wulff and Zhorov, 2008). ShK and derivatives reduce the inflammatory response of autoimmune diseases, by maintaining the integrity of BBB (Huang et al., 2017), reducing activation of TEM cells (Beeton et al., 2006), and eliminating respiratory bursts in activated microglia and subsequent secondary damage of neurons by microglia (Fordyce et al., 2005). Interestingly, experiments have demonstrated that this inhibition is achieved by effectively inhibiting the proliferation of TEM cells without affecting naive or TCM lymphocytes.

ShK-170 also plays an effective role in preventing active delayed type hypersensitivity (DTH) and acute adoptive EAE as well as in treating acute adoptive EAE in rats (Beeton et al., 2005). ShK-186 could inhibit DTH, TEM cell enlargement (Tarcha et al., 2012) and movement in inflamed tissues, but had no effect on lymph node homing or movement in naive and central memory T (TCM) cells (Matheu et al., 2008). ShK-186 can effectively improve the symptoms in a rat model of MS with good safety (Matheu et al., 2008).

In addition to ShK and its analogs, ChTX is specific to both KCa3.1 and Kv1.3 channels in human T lymphocytes, but the expression level of KCa3.1 in TEM is very low (Hu et al., 2007). Therefore, it is most likely to inhibit the proliferation of TEM cells by effectively blocking the Kv1.3 channel. ChTX might be a potential drug for MS. BmKTX-D33H inhibits cytokine production and proliferation in human T cells *in vitro* and significantly improves delayed hypersensitivity (DTH) response (Chen et al., 2018), highlighting its advantages as a potential drug for autoimmune diseases. Moreover, oligodendrocyte (OLG) causes axonal myelination of the CNS (Trapp et al., 1997) and the complement complex (C5b-9, composed of C5b, C6, C7, C8, and C9 proteins) is capable of inducing cell cycle activation in OLGs (Rus et al., 2006). Studies have found that complement activation and destruction of OLGs are among the most common pathological forms of MS (Cudrici et al., 2006). It is worth noting that Kv1.3 plays an important role in this process (Tegla et al., 2011). This explains the potential value of Kv1.3 blockers including rOsK-1 (Tegla et al., 2011) in the treatment of MS.

### Ischemic Stroke

Stroke is an acute cerebrovascular disorder that causes brain tissue damage, which is the second leading disease causing sudden death after ischemic heart disease and accounts for 9% of deaths worldwide (Schmitz et al., 2005). Ischemic stroke is the most common type of stroke, usually occurring when the blood vessels in the neck or brain are blocked (Wallace et al., 2016). Formation of a clot in the blood vessels of the brain or neck, followed by its translocation to other part of the body, such as the heart, to the brain, which may cause severe narrowing of the arteries in the brain or to the brain, results in a stroke (van Os et al., 2016).

In the early stages of stroke, activated macrophages (M1) release a variety of cytokines (TNF-α, IL-1-β, and IL-23), trigger neuronal damage, and induce TEM cell-mediated further inflammatory responses (Man et al., 2017). A few days later, macrophages could change to M2-like functions and begin to clear various inflammatory factors, cell debris, and secreted anti-inflammatory as well as neurotrophic factors (IL-10, TGF-β, and IGF-1) to promote injury recovery (Man et al., 2017).

Similarly, microglia are highly malleable and can exhibit different phenotypes depending on different micro-environmental signals. Lipopolysaccharide (LPS) and IFN-γ promote the differentiation of microglia into classical activated M1 type, along with producing high levels of pro-inflammatory cytokines, nitric oxide, and continuously impairing the CNS parenchyma (Huang et al., 2017), which contribute to the secondary expansion of the infarct (Chen et al., 2018).

The voltage-gated potassium channel Kv1.3 plays important roles in microglia as well as macrophage activation by modulating Ca^2+^ signaling, oxidative burst, cytokine production, and neuronal killing (Kirk et al., 2003; Peelen et al., 2011; Ortiz et al., 2014), which is required for microglia or macrophage M1-like pro-inflammatory activation *in vivo* (Di Lucente et al., 2018) (Figure 2). Activated microglia in the pathology of ischemic stroke significantly contributes to secondary expansion of the infarct, and Kv1.3 blockers are thought to be useful in ameliorating this condition (Iadecola and Anrather, 2011; Macrez et al., 2011). Studies have shown that Kv1.3 inhibitors can retain beneficial “M2-like” functions while preferentially inhibiting “M1-like” inflammatory microglia/macrophage function (Murray et al., 2014). The most effective and specific small molecule, PAP-1 can inhibit Kv1.3 at an IC_50_ of 2 nmol/L (Schmitz et al., 2005), is orally available, brain penetrant, and does not have any long-term toxicity in rodents or primates (Azam et al., 2007; Pereira et al., 2007). It is worth noting that PAP-1 significantly reduces the levels of pro-inflammatory cytokines and infarct volume after ischemic injury and improves neurological deficits (Chen et al., 2018).

We speculate that other ShK derivatives are also likely to reduce pro-inflammatory factors and improve brain damage by inhibiting M1-like function of microglia or macrophages. It was reported that ShK-170, a compact derivative of ShK-186, has been shown to protect mice from microglia-mediated radiation-induced brain damage (Peng et al., 2014). Therefore, Kv1.3-specific inhibitor ShK could be expected to be potential novel therapeutic agents for acute ischemic stroke. In addition to its significant role in immunity and inflammation, Kv1.3 has also found its potential role in the coagulation system (Ding et al., 2018). BF9 is a Kv1.3 blocker with anticoagulant activity (Ding et al., 2018) and provides a new molecular template for the discovery of lead drugs for immune and thrombotic-related human diseases.

### Alzheimer’s Disease

Alzheimer’s disease (AD) is a progressive neurodegenerative disorder of the brain, which is characterized by the structural and functional loss of neurons. The main pathogenesis now known has been described as progressive proliferation of plaques outside neurons (extracellular amyloid plaques) and neurofibrillary tangles inside neurons (hyperphosphorylated tau accumulation) (Takahashi et al., 2010; Selkoe and Hardy, 2016). However, a large number of amyloid reduction therapy (ART) clinical trials did not find the expected clinical improvement in AD patients. More and more attention has been paid to the neuroinflammation cascades mediated by primed microglia cells contributing to AD pathogenesis (Krause and Muller, 2010). Studies have shown that cytotoxic substances and pro-inflammatory cytokines, secreted by activated microglia, could induce nerve damage and aggravate the pathology of AD (Heneka et al., 2015). The modules underlying immune microglial cells or T lymphocytes as molecular systems were closely related to the pathophysiology of AD, which were based on a network-based analysis of whole-genome gene-expression profiling and genotypic data obtained from 1647 AD as well as non-demented brain samples (Zhang et al., 2013). Neuroinflammation cascades mediated by activated microglial cells and T lymphocytes contribute to AD pathogenesis (Janke and Yong, 2006; Zheng et al., 2008). Thus, it is concluded that immune-associated alterations significantly contribute to the pathophysiology of AD, even though an effective therapeutic target is not yet available in clinic.

In addition to being a drug target for arrhythmias and Type 2 diabetes, K^+^ channels have also been proposed as targets for the treatment of immunosuppression, cancer, and various neurological disorders. Including the abnormal expression of K^+^ channel has been detected in brains of AD patients (Poulopoulou et al., 2010). Furthermore, the ShK-sensitive Kv1.3 channel is mainly expressed in T lymphocytes, macrophages, and microglia, and the up-regulated expression of Kv1.3 channels in human T and B lymphocytes was closely related to the occurrence and development of autoimmune diseases (Jang et al., 2015; Kazama, 2015). Microglia represent innate immune cells in AD that mediate neuroinflammation, and voltage-gated Kv1.3 potassium channels are key regulators of microglial function. Previous study has showed that Kv1.3 plays an important role in immune cell activation by modulating Ca^2+^ signaling (Feske et al., 2015) (Figure 2) and in the AD model through the high expression of pro-inflammatory microglia (Maezawa et al., 2018). Moreover, the expression of Kv1.3 is increased in human AD brains and the elevation is limited to microglial cells. Kv1.3 blockers have further been demonstrated to inhibit the activation of microglia, which could mediate neurotoxicity in cell culture (Fordyce et al., 2005). These findings suggest that Kv1.3 might be a pathologically relevant microglial target in AD (Rangaraju et al., 2015). Similarity, the Kv1.3 channel was also found to be expressed in T lymphocytes (Chandy et al., 1984; DeCoursey et al., 1984). With continued investigation in AD patients, high expression levels of Kv1.3 channels were successively confirmed in activated T cell (Beeton et al., 2006). Kv1.3 channel blockers were able to inhibit TEM cell activation and suppress the secretion of related cytokines (such as IL-2, IL-4, IFN-γ, and TNF-α) (Beeton et al., 2006; Nicolaou et al., 2007). In summary, the microglial and lymphocytic Kv1.3 channels are becoming an attractive target for the research and generation of drugs against ADs.

At present, the number of *in vivo* diagnostic techniques available for detection of AD is very limited. Several drugs for treating symptoms of AD do not alter disease progression and their benefits are at most modest, which indicates an urgent need for novel target discovery. As mentioned above, the expression of Kv1.3 is increased in AD brains and blocking its expression might be beneficial for AD patients. Pro-inflammatory disease-associated microglia (DAM) emerged early in the AD mouse model and was characterized by a pro-inflammatory gene (Il12b, Ilb), with surface marker CD44, potassium channel Kv1.3, and regulatory factors. It was reported that the ShK-223 peptide promotes anti-inflammatory DAM by agonizing LXRα/β and blocking Kv1.3, inhibits pro-inflammatory DAM, and increases Aβ clearance in the AD model (Rangaraju et al., 2018). Subsequently, Norton and Chandy (2017) investigated the effects of BmKTX (ADWX-1), OsK1-K16-D20, and HsTx1 [R14A] targeting Kv1.3 in the treatment of AD (Norton and Chandy, 2017).

Studies showed that PAP-1, as a small molecule Kv1.3 blocker, could reduce neuroinflammation, decrease cerebral amyloid load, enhance hippocampal neuronal plasticity, and improve behavioral deficits in APP/PS1 transgenic mice (Maezawa et al., 2018). Thus, we infer that using Kv1.3-specific blockers as attractive therapeutic agents to mitigate Aβ-induced pro-inflammatory microglia, which are highly relevant to AD pathogenesis. Therefore, ShK and its analogs, which were the specific blockers of Kv1.3, are capable to be the candidate drugs for AD.

### Other Pathologies

The activation of voltage-gated potassium channels after the action potential is the main regulatory effect that determines the degree of repolarization and repeated neuronal discharge. Epilepsy is a chronic disease of transient brain dysfunction caused by sudden abnormal discharge of brain neurons (Wittner et al., 2001; Manford, 2017). Potassium channels play an important role in maintaining resting membrane potential and regulating cell excitability of neurons, which can cause neuropathic pain or neurological diseases (such as epilepsy and ataxia) (Wickenden, 2002; Pimentel et al., 2008). In the Kv channel, the Kv1 subfamily leads to differences in susceptibility to epilepsy in the brain (Pena and Coimbra, 2015). In fact, many of the symptoms of hyperexcitability, including epilepsy, are caused by mutations or downregulation of the Kv1 channel (Glasscock, 2019; Verdura et al., 2019). Studies have shown that the convulsive agent pentylenetetrazol can significantly reduce the Kv1.3 currents (Madeja et al., 1997), indicating that Kv1.3 may be associated with epileptogenesis (Figure 2). Activation of microglia and neuroinflammation are important markers of epileptogenesis (Eyo et al., 2017). The activation of microglia and the expression of inflammatory factors were also positively correlated with the progression of epilepsy in the hippocampus of patients with epilepsy (Hiragi et al., 2018). Early studies have shown that pro-inflammatory substances induce the increase of potassium currents in microglia (Fordyce et al., 2005). The presence of Kv1.3 in microglia regulates the proliferation of glial cells, with causing potassium efflux (Fordyce et al., 2005; Peng et al., 2014). The above data indicate that abnormal expression of Kv1.3 is closely related to the occurrence and development of epilepsy.

Parkinson’s disease (PD) is a degenerative disease of the CNS, which involves motor deficits including tremors, muscle rigidity, bradykinesia, and impaired gait (Sampson et al., 2016). Tubert et al. (2016) showed that Kv1.3-mediated currents in PD striatum significantly reduced the inhibition of cholinergic interneuron excitability. Early studies have found that microglia are activated in the early stages of PD patients and throughout the disease process (Sanchez-Guajardo et al., 2015). The upregulation of K^+^ channels is considered as a hallmark of microglial activation (Rangaraju et al., 2015). Upon this activation, there is an increased expression of Kv channels (Di Lucente et al., 2018), mainly Kv1.5 and Kv1.3 channels. Among them, Kv1.3 currents become predominant upon proliferation of microglial cells (Kotecha and Schlichter, 1999). Accumulated data indicate that Kv1.3 channels maybe as potential targets for PD therapy (Figure 2).

The above fact suggests that ShK, as an effective regulator of microglia and T lymphocyte activation (Castaneda et al., 1995), and Kv1.3 blockers, has a high pharmacological value for the development of a more stable and highly selective ShK. They constitute a large pharmacological armamentarium to target Kv1.3 channels with high potency and specificity (Wulff and Zhorov, 2008), which could offer treatments targeting epilepsy and PD. In addition, MgTx (Bartok et al., 2014), AgTx-2 (Pimentel et al., 2008), OsK-1 (Mouhat et al., 2005), and BF9 (Chen et al., 2001) depend on their functional characteristics that can effectively inhibit Kv1.3, which have medical values on the further pharmacological studies.

## Prospect and Conclusion

Up to now, there are 15 venom-derived drugs that used to treat a variety of diseases, including hypertension, pain, and diabetes, in clinic. As a result, many lives have been saved. Moreover, 13 animal-derived toxins are considered to be drug candidates, having been entering in clinical trials (King, 2011). Among them, ShK derivatives, ShK-186 and ShK-192, mainly used to treat autoimmune diseases, including neuroinflammatory MS by targeting Kv1.3 channels. In this review, we discuss the possibility of ShK for clinical treatment on other Kv1.3 relevant neuroinflammatory diseases. It is shown that ShK could effectively suppress the activation of microglial cells as well as T lymphocytes in stroke, epilepsy, AD, and PD. *In vivo* studies also demonstrated that inhibition of Kv1.3 is favorable for the reversion of neuroinflammatory diseases. This brings the dawn of effective control of diseases such as AD and PD that are suspected to be overcome. However, it is still a challenge for ShK used to the treatment of neuroinflammatory diseases. The first problem underlying the application of these peptides is that they couldn’t be taken orally, mainly because they are difficult to penetrate the intestinal mucosa. Due to the molecular size, polarity, hydrophilicity, and chargeability, the cell membrane penetration of ShK is hampered. The second obstacle is that ShK cannot cross the BBB. Different from MS, the myelin and BBB are not destroyed in other neuroinflammatory diseases (Li et al., 2015). Clinical application of ShK for treating neuroinflammatory diseases will encounter difficulties. Fortunately, the situation is not unsolvable, we still have a glimmer of light. A few years ago, scientists at the Sunnybrook Health Science Center in Canada used focused ultrasound technology to successfully pass chemotherapy drugs across the BBB in a non-invasive manner (Burgess et al., 2015) and reach the location of the tumor, which is of great significance in the field of neuropharmacology. In addition, the cell penetrating peptide (CPP) (Kristensen et al., 2016) with a strong cell membrane penetration could be used as a drug carrier to assist the passage of polypeptide drugs across the cell membrane (Li et al., 2015). The fusion protein consists of CPP and ShK might be developed as an oral drug for neuroinflammatory diseases. In short, finding a suitable, safe, and efficient way to promote the clinical use of ShK is the most valuable points to be solved.

## Author Contributions

XW, GL, ZZ, SZ, YZ, YJ, and JT drafted the manuscript and revised it critically for intellectual content. JG, LY, and JC drawn the figures. All authors read and approved the final version of the manuscript before submission.

## Conflict of Interest

The authors declare that the research was conducted in the absence of any commercial or financial relationships that could be construed as a potential conflict of interest.

## Footnotes

1 https://www.rcsb.org/
