# Corrigendum: Kv1.3 Channel as a Key Therapeutic Target for Neuroinflammatory Diseases: State of the Art and Beyond

**DOI:** 10.3389/fnins.2020.00163

**Published:** 2020-02-28

**Authors:** Xiaoli Wang, Guoyi Li, Jingkang Guo, Zhiping Zhang, Shuzhang Zhang, Yudan Zhu, Jiwei Cheng, Lu Yu, Yonghua Ji, Jie Tao

**Affiliations:** ^1^Department of Neurology and Central Laboratory, Putuo Hospital, Shanghai University of Traditional Chinese Medicine, Shanghai, China; ^2^Institute of Biomembrane and Biopharmaceutics, Shanghai University, Shanghai, China; ^3^Xinhua Translational Institute for Cancer Pain, Shanghai, China; ^4^Putuo Clinical Medical School, Anhui Medical University, Shanghai, China

**Keywords:** Kv1.3, ShK, neuroinflammatory disease, multiple sclerosis, stroke, epilepsy, Alzheimer's disease, Parkinson's disease

In the original article, we neglected to include the funder **Youth Talent Promotion Project of China Association of Chinese Medicine**, **grant no. 2019-QNRC2-C10** to **Jie Tao**. In addition, the order of the affiliations was incorrect. Affiliations 1 and 2 have been switched and the change is reflected above.

The authors apologize for this error and state that this does not change the scientific conclusions of the article in any way. The original article has been updated.

